# MXS-Chaining: A Highly Efficient Cloning Platform for Imaging and Flow Cytometry Approaches in Mammalian Systems

**DOI:** 10.1371/journal.pone.0124958

**Published:** 2015-04-24

**Authors:** Hanna L. Sladitschek, Pierre A. Neveu

**Affiliations:** Cell Biology and Biophysics Unit, European Molecular Biology Laboratory, Heidelberg, Baden-Württemberg, Germany; H. Lee Moffitt Cancer Center & Research Institute, UNITED STATES

## Abstract

The continuous improvement of imaging technologies has driven the development of sophisticated reporters to monitor biological processes. Such constructs should ideally be assembled in a flexible enough way to allow for their optimization. Here we describe a highly reliable cloning method to efficiently assemble constructs for imaging or flow cytometry applications in mammalian cell culture systems. We bioinformatically identified a list of restriction enzymes whose sites are rarely found in human and mouse cDNA libraries. From the best candidates, we chose an enzyme combination (**M**luI, **X**hoI and **S**alI: MXS) that enables iterative chaining of individual building blocks. The ligation scar resulting from the compatible XhoI- and SalI-sticky ends can be translated and hence enables easy in-frame cloning of coding sequences. The robustness of the MXS-chaining approach was validated by assembling constructs up to 20 kb long and comprising up to 34 individual building blocks. By assessing the success rate of 400 ligation reactions, we determined cloning efficiency to be 90% on average. Large polycistronic constructs for single-cell imaging or flow cytometry applications were generated to demonstrate the versatility of the MXS-chaining approach. We devised several constructs that fluorescently label subcellular structures, an adapted version of FUCCI (fluorescent, ubiquitination-based cell cycle indicator) optimized to visualize cell cycle progression in mouse embryonic stem cells and an array of artificial promoters enabling dosage of doxycyline-inducible transgene expression. We made publicly available through the Addgene repository a comprehensive set of MXS-building blocks comprising custom vectors, a set of fluorescent proteins, constitutive promoters, polyadenylation signals, selection cassettes and tools for inducible gene expression. Finally, detailed guidelines describe how to chain together prebuilt MXS-building blocks and how to generate new customized MXS-building blocks.

## Introduction

The discovery of green fluorescent protein (GFP) has enabled biologists to monitor complex processes like gene expression, cell migration or subcellular protein localization in living cells. The ever-expanding palette of fluorescent proteins and the continuous improvement of imaging technologies have driven the demand for customized fluorescent reporters. Assembly of such constructs should ideally be robust, time efficient, intuitive to design and flexible enough to allow for the incremental optimization of prototypes. Several cloning strategies have been devised that represent different trade-offs between these criteria.

The Golden Gate method uses type II restriction enzymes to seamlessly assemble up to 10 modules in a single step [1]. Hierarchical Golden Gate shuffling iterates this shuffling process to bypass the limitation on the number of modules that can be assembled into the final construct. This approach offers an ideal solution for generating combinatorial libraries, in which one or a few modules are to be varied. However, the assembly process imposes a stringent constrain on the order of elements to be combined as neighboring fragments require compatible cohesive ends. The generation of permutational combinations necessitates therefore very large libraries of modules (see [2] for example). It is furthermore cumbersome to design optimized hierarchical assembly trees that produce intermediates, which can be recycled for the assembly of other constructs.

Gibson cloning enables the seamless combination of several PCR products in a single reaction without the need for restriction enzymes: an exonuclease produces fragments with single-stranded ends, which anneal to complementary ends of neighboring elements, a DNA polymerase fills the gaps and a ligase covalently joins the neighboring fragments [3]. Gibson cloning has been extremely successful in assembling large DNA sequences. However, it is ill suited to join together sequences that share a high degree of identity making it difficult to generate polycistronic constructs (with e.g. multiple identical polyadenylation signals or promoters).

Chaining-based methods, like BioBricks, are well suited for the modular assembly of highly similar or repetitive sequences [4]. The BioBricks method combines two modules using restriction enzymes that generate compatible cohesive ends from two different recognition sites, whose ligation scar (i.e. the sequence generated by the compatible overhangs upon ligation) is not recognized by either of the employed enzymes. The original pattern of restriction sites is therefore regenerated in the ligation product, which hence forms a new BioBrick part. Intermediate chaining products that perform a certain task are called cassettes and can be readily reused in the assembly of any later construct. In our experience the recyclability of functional cassettes, the intuitive, highly flexible construct design and the robustness of cloning make chaining-based approaches an excellent choice for routine applications and outweigh the immediate time-savings of one-pot assembly strategies in the long run.

As chaining-based methods can only work if the restriction sites used for the assembly are not found within the modules themselves (or have been removed by mutagenesis), the choice of restriction enzymes is critical to guarantee the wide applicability of the approach. While BioBrick assembly responds to the need for flexible, modular cloning strategies, little attention has been invested to facilitate fusion of endogenous coding sequences to protein tags or fluorescent proteins. In fact, unfavorable enzyme choices have hampered the application of chaining-based cloning approaches to mammalian cell culture systems and some standards do not support in-frame fusions of coding sequences.

Here we describe a chaining method that is specifically designed for the assembly of constructs for imaging and flow cytometry approaches in mammalian cell culture systems. We offer a comprehensive library of parts and cassettes through the Addgene repository (https://www.addgene.org) to allow researchers to specifically tailor constructs to their needs. To demonstrate the versatility of the technique, we engineered several constructs that fluorescently label cellular landmarks, an adaptation of the fluorescent, ubiquitination-based cell cycle indicator (FUCCI) [5] to mouse embryonic stem cells and an array of artificial doxycyline-inducible promoters [6].

## Methods

### Prediction of restriction enzymes

We developed a custom script in Python using the Biopython module. Mouse, human, *Drosophila* and zebrafish cDNA sequences were downloaded from Ensembl. From the New England Biolabs set of restriction enzymes, we kept only 6-cutters, removed blunt-end cutters and enzymes with degenerate recognition sequences. We then counted for each enzyme how many restriction sites were found in the cDNA sequences.

### Preparation of backbones

Sequences were analyzed and annotated using the Geneious version 5.6.7 [7].

All building blocks were assembled into small customized vector backbones (≈1.9 kb) based on puC19 (Genbank L09137, [8]) containing the *β*-lactamase gene conferring resistance to ampicillin and a high copy origin of replication. The minimal backbone was generated by PCR on pUC19 plasmid using primers (pUC19_F: 5’-AGGTACCATG**GCTCACTGACTCGCTGCGCT**-3’ and pUC19_R: 5’-AGGTACCTTGAAT**TCGGGGAAATGTGCGCGGAA**-3’) followed by KpnI-digest, gel purification and self-ligation. To generate the MXS_chaining vector a multiple cloning site containing EcoRV-MluI-XhoI-SalI restriction sites was generated by inserting the annealed oligos (MXS_F: 5’-CATGATATCAAACGCGTAACCTCGAGAACGTCGACC-3’ and MXS_R: 5’-AATTGGTCGACGTTCTCGAGGTTACGCGTTTGATAT-3’) into the NcoI-, EcoRI-digested minimal backbone. To generate the MXS_MCS1 vector a multiple cloning site containing EcoRV-MluI-XhoI-NheI-XbaI-NcoI-KpnI-BclI-BamHI-SalI restriction sites was generated by inserting the annealed oligos (MCS1_F: 5’-CATGATATCAAACGCGTAACTCGAGGCTAGCTATATCTAGATATACCATGGTATAGGTACCTATATGATCATATAGGATCCGTCGACC-3’ and MCS1_R: 5’-AATTGGTCGACGGATCCTATATGATCATATAGGTACCTATACCATGGTATATCTAGATATAGCTAGCCTCGAGTTACGCGTTTGATAT-3’) into the NcoI-, EcoRI-digested minimal backbone. To generate the MXS_MCS2 vector a multiple cloning site containing EcoRV-MluI-XhoI-EcoRI-BglII-KpnI-SalI restriction sites was generated by inserting the annealed oligos (MCS2_F: 5’-CATGATATCAAACGCGTAACTCGAGGAATTCAAAGATCTTCGGTACCGTCGACC-3’ and MCS2_R: 5’-AATTGGTCGACGGTACCGAAGATCTTTGAATTCCTCGAGTTACGCGTTTGATAT-3’) into the NcoI-, EcoRI-digested minimal backbone. To generate the MXS_MCS3 vector a multiple cloning site containing MluI-XhoI-HindIII-PstI-SalI restriction sites was generated by inserting the annealed oligos (MCS3_F: 5’-CATGACGCGTAACTCGAGAAGCTTAATCCTGCAGGTCGAC-3’ and MCS3_R: 5’-AATTGTCGACCTGCAGGATTAAGCTTCTCGAGTTACGCGT-3’) into the NcoI-, EcoRI-digested minimal backbone. To generate the MXS_MCS4 vector a multiple cloning site containing MluI-XhoI-PvuII-BglII-SalI restriction sites was generated by inserting the annealed oligos (MCS4_F: 5’-CATGACGCGTAACTCGAGGCAGCTGAGATCTGTCGAC-3’ and MCS4_R: 5’-AATTGTCGACAGATCTCAGCTGCCTCGAGTTACGCGT-3’) into the NcoI-, EcoRI-digested minimal backbone. To generate the SMX_DEST vector a multiple cloning site containing SalI-EcoRV-MluI-XhoI restriction sites was generated by inserting the annealed oligos (SMX_F: 5’-CATGAGTCGACAACTGATATCATAACGCGTAATCCTCGAGC-3’ and SMX_R: 5’-AATTGCTCGAGGATTACGCGTTATGATATCAGTTGTCGACT-3’) into the NcoI-, EcoRI-digested minimal backbone.

The identity and integrity of all newly cloned building blocks were confirmed by Sanger sequencing using the primers seq_F: 5’-TTACCGCCTTTGAGTGAG-3’ and seq_R 5’-TTGTCTCATGAGCGGATAC-3’ which bind inside the minimal backbone on either side of the respective MCS.

### Preparative restriction digest

Restriction enzymes MluI (R0198), SalI-HF (R3138) and XhoI (R0146) were from NEB. Buffer 3.1 (B7203; final concentration: 100 mM NaCl, 50 mM Tris-HCl, 10 mM MgCl_2_, 100 *μ*g/ml BSA, pH 7.9) was used for MluI-, SalI- and MluI-, XhoI-double-digests. 1 *μ*g of plasmid DNA or PCR product was incubated with 5 U of each restriction enzyme in a final volume of 30 *μ*l for 1 h at 37°C. DNA fragments were separated on 0.8% Agarose (Sigma) gels and purified using the QIAquick gel purification Kit (Qiagen).

### Annealing reactions

Pairs of oligonucleotides (Sigma) were mixed at a final concentration of 50 *μ*M each in 10 mM Tris-HCl (pH 8.5) in a total volume of 50 *μ*l. The tubes were placed into a beaker with boiling water, which was allowed to cool to RT. The annealed oligonucleotides were diluted 1:100 in 10 mM Tris-HCl (pH 8.5) and 1 *μ*l of the dilution (0.5 pmol) was directly used for ligation.

### PCR conditions

Primers were purchased from Sigma. For PCR reactions with plasmid DNA as a template the following reaction condition were used: 0.25 *μ*l forward primer (100 *μ*M), 0.25 *μ*l reverse primer (100 *μ*M), 30 ng plasmid DNA template, 10 *μ*l Phusion HF buffer (NEB) (5x), 1.5 *μ*l DMSO (NEB), 1 *μ*l dNTPs (NEB) (10 mM each), *ad* 50 *μ*l ddH_2_O. Just before starting the reaction, 0.5 U Phusion high-fidelity DNA polymerase (NEB) were added. The following PCR program was used (on a S-1000 Thermal Cycler, BioRad): initial denaturation (98°C, 30 sec), 8x [denaturation (98°C, 10 sec), annealing (62°C, 15 sec), elongation (72°C, 20 sec per kb)], 18x [denaturation (98°C, 10 sec), annealing & elongation (72°C, 30 sec per kb], final extension (72°C, 5 min). For PCR amplification from cDNA, 1 *μ*l of cDNA (out of a total reaction volume of 20 *μ*l) served as template. The following PCR program was used: initial denaturation (98°C, 2 min), 40x [denaturation (98°C, 15 sec), annealing (gradient temperatures from 56 to 66°C, 30 sec), elongation (72°C, 30 sec per kb)], final extension (72°C, 5 min). PCR products were separated on 0.8% Agarose (Sigma) gels and purified using the QIAquick gel purification Kit (Qiagen). After MluI-, SalI-restriction digest, enzymes were removed using the QIAquick PCR purification kit and the digested fragments were used for ligation.

### Site-directed mutagenesis

Mutagenesis primers were designed using the Quikchange Primer Design Program (Agilent). The following reaction conditions were used: 0.6 *μ*l forward primer (100 ng/*μ*l), 0.25 *μ*l reverse primer (100 ng/*μ*l), 30 ng template, 5 *μ*l Phusion HF buffer (NEB) (5x), 0.75 *μ*l DMSO (NEB), 0.5 *μ*l dNTPs (NEB) (10 mM each), *ad* 25 *μ*l ddH_2_O. Just before starting the reaction, 0.5 U Phusion high-fidelity DNA polymerase (NEB) were added. The following PCR program was used: initial denaturation (98°C, 2 min), 18x [denaturation (98°C, 20 sec), annealing (60°C, 20 sec), elongation (72°C, 30 sec per kb)], final extension (72°C, 2 min). 5 U DpnI (NEB) were added and the reaction was incubated 37°C for 1 h. 5 *μ*l of the reaction were transformed into competent MachT1 cells following the protocol described below.

### Ligation

For a typical ligation, 1 *μ*l of gel elution of the cut backbone, 8 *μ*l of gel elution of the cut insert, 1 *μ*l of T4 DNA Ligase Reaction Buffer (10X) (NEB), and 100 U T4 DNA Ligase (NEB) were mixed and the reaction was incubated at RT for 15 min.

### Transformation

Chemically competent MachT1 (Invitrogen) were prepared using the calcium-manganese-based (CCMB) method as described [9]. 5 *μ*l of the ligation reaction were added to 50 *μ*l of competent bacteria thawed on ice. The DNA-bacteria mixture was incubated on ice for 2 min, heat shocked (42°C, 1 min), placed back on ice (30 sec), and plated directly on ampicillin selection plates. For plasmids > 10 kb, bacteria were allowed to recover in 1 ml SOC (2% tryptone, 0.5% yeast extract, 10 mM NaCl, 2.5 mM KCl, 10 mM MgCl_2_, 10 mM MgSO_4_, 20 mM glucose) at 37°C for 40 min (150 rpm) prior to plating.

### Cell culture and establishment of stable mESC cell lines

Mouse ESCs E14tga2 [10] were maintained without feeders in “LIF+serum” medium composed of DMEM (high glucose, no glutamine, with sodium bicarbonate, Invitrogen) supplemented with 15% (v/v) ES-qualified fetal calf serum (EmbryoMax, Millipore), 10 ng/ml murine LIF (EMBL Protein Expression and Purification Core Facility), 1x non-essential amino acids, 2 mM L-glutamine, 1 mM sodium pyruvate, 100 U/ml penicillin and 100 *μ*g/ml streptomycin, 0.1 mM *β*-mercaptoethanol (all Invitrogen) on culture dishes (Nunc) coated with 0.1% (v/v) gelatin (Sigma) solution and cultured at 37°C with 5% CO_2_. Medium was changed daily and cells were passaged every other day with 0.05% Trypsin-EDTA (Invitrogen) at a passaging ratios of 1/6–1/12. For generation of stable cell lines, plasmids were linearized and transfected using Fugene HD (Promega) according to the manufacturer’s protocol. After antibiotic selection single colonies were expanded and screened for expression of the transgene. Transgenic lines were karyotyped as described [11].

HeLa Kyoto cells [12] were maintained in DMEM (high glucose, no glutamine, with sodium bicarbonate, Invitrogen) supplemented with 10% (v/v) fetal calf serum (Millipore), 1x non-essential amino acids, 2 mM L-glutamine, 1 mM sodium pyruvate, 100 U/ml penicillin and 100 *μ*g/ml streptomycin (all Invitrogen) on culture dishes (Nunc) and cultured at 37°C with 5% CO_2_.

### Flow cytometry

Cells were dissociated to single-cell suspension with 0.05% Trypsin-EDTA (Invitrogen), resuspended in D-PBS, strained through a 40 *μ*m cell strainer (BD Biosciences) and analyzed on an LSRFortessa flow cytometer (BD BioSciences). Flow cytometry data was gated on forward and side scatters using FlowJo software and further analyzed using custom Python scripts.

### Live imaging

Transgenic mESCs were seeded at a density of 2000 cells/cm^2^ onto gelatinized glass-bottom dishes. HeLa cells were seeded onto glass-bottom dishes and transfected with Lipofectamine 2000 (Invitrogen) at 80% confluency according to the manufacturer’s instructions one day before image acquisition. Images were acquired on an inverted SP8 confocal microscope (Leica) equipped with 40x PL Apo 1.1 W objective in an incubation chamber at 37°C under a 5% CO_2_ atmosphere and 80% relative humidity. TagBFP, Cerulean, mAzamiGreen, Citrine, mCherry and iRFP670 were excited with the 405 nm, 458 nm, 488 nm, 514 nm, 561 nm and 633 nm lasers, respectively. Kaede was photoconverted using the 405 nm laser. The green-emitting Kaede state was excited with the 488 nm laser, the red-emitting state with the 488 nm and 561 nm lasers. Channels were acquired sequentially using HyD detectors.

## Results and Discussion

### Identification of chaining enzymes

We aimed to identify a set of restriction enzymes generating compatible cohesive ends, which i) cut as rarely as possible in human and mouse cDNAs, are ii) affordable and reliable in their cutting performance and possess compatible reaction condition preferences, and iii) whose ligation will generate a translatable scar. We downloaded the Ensembl human and mouse cDNA libraries and counted the number of restriction sites for all 6-cutters (i.e. enzymes with recognition sequences that are specified by a 6-mer of nucleotides and whose ligation scar will hence be equivalent to two codons when translated) available from the largest commercial supplier of restriction enzymes (New England Biolabs). SalI and XhoI formed the best pair and MluI was the best choice for the direction-giving enzyme. This enzyme set was compared to the ones used by two common chaining approaches. The Biobrick strategy uses EcoRI and PstI as direction giving enzymes and SpeI and XbaI for chaining [4]. The Bglbricks strategy uses EcoRI as direction giving enzymes and BglII and BamHI for chaining [13]. In human cDNAs, MluI had 7.57 and 16.7 fewer sites than EcoRI and PstI respectively. SalI had 4.9 and 1.8 times fewer sites than BamHI and SpeI respectively. XhoI has 2.1 and 1.35 times fewer sites than BglII and XbaI respectively. We obtained similar numbers when using mouse cDNAs. Thus the enzyme set we determined was better suited to human and mouse applications than the ones used in common standards. Interestingly, the enzyme pair was different for non-mammalian species. For example, SpeI and XbaI were better suited for the fruitfly (*Drosophila melanogaster*), and NheI and SpeI for zebrafish (*Danio rerio*). Those results can be understood in light of CpG dinucleotide frequency. Indeed CpG dinucleotides have long been known to be underrepresented in most vertebrate genomes [14, 15]. This explains why SalI, XhoI and MluI recognition sites, which contain one, one and two CpG dinucleotides in their 6-mer sequences, are rarely found in the transcriptome of these species. As an outlier to that rule, fish genomes are characterized by a higher frequency of CpG dinucleotide [16], which is reflected by the difference in the optimal enzyme set.

Importantly, the three enzymes MluI, XhoI and SalI can be used in the same reaction conditions. Because XhoI had roughly three times more sites in mouse cDNAs compared to SalI, we chose to flank the building blocks with MluI and SalI. This enabled us to subclone new building blocks containing an XhoI site using MluI-, SalI-digest and to remove the internal XhoI site only after subcloning by site-directed mutagenesis.

### The MXS-chaining standard

**Fig 1:**
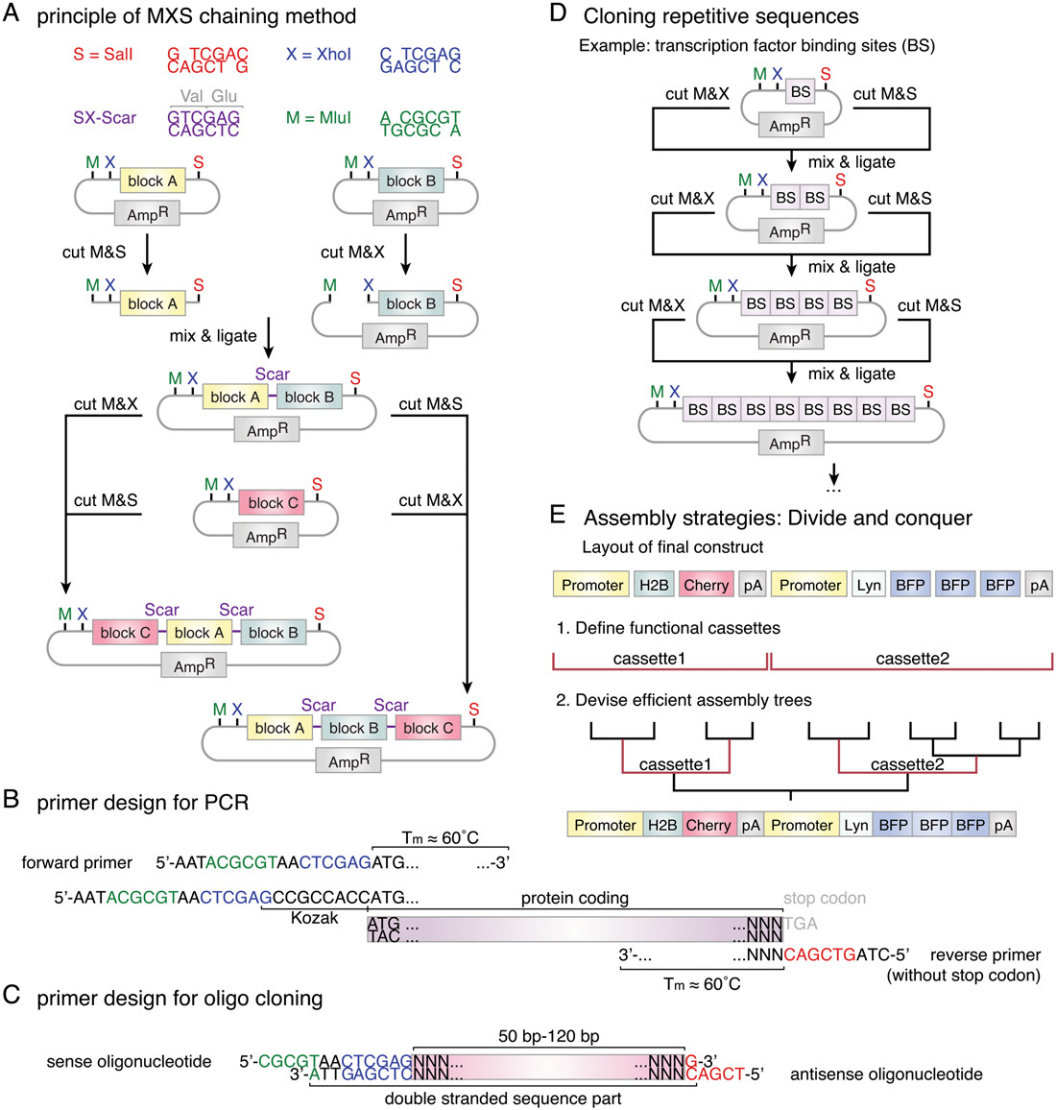
Scheme of the MXS-chaining strategy. (A) MluI (M), XhoI (X) and SalI (S) recognition sites are highlighted in green, blue and red, respectively. SalI- and XhoI-overhangs form a compatible pair whose cohesive ends give rise to a translatable scar (coding for valine and glutamic acid) upon ligation. Individual building blocks can be chained together in the desired order, with the MS-digested building block forming the 5’-part and the MX-digested building block the 3’-part of the new chaining module. As the XS-Scar is not cut by any of the restriction enzymes used in the chaining system the original pattern of restriction sites is recreated, which allows the reiteration of the chaining procedure. (B) New building blocks can be isolated from plasmid, genomic or cDNA by PCR. The melting temperature (Tm) of the template binding part of pairs of primers should not differ by more than 1K and be close to 60°C. 5’-extensions on forward and reverse primers enable direct subcloning of the PCR product into the MXS-chaining context. For protein coding sequences, the maintenance of the open reading frame has to be assured and the potential addition of a Kozak sequence should be considered. (C) Small building block sequences (< 120 bp) can be directly cloned from annealed complementary oligonucleotides with MluI- and SalI-overhangs. (D) MXS-chaining allows to quickly generate building blocks harboring any desired number of repeats of a specific sequence motif. This feature is of particular interest for the generation of transcriptional reporters as the number of incorporated transcription factor binding sites can be readily optimized. (E) Several chaining steps needed for the completion of a construct can be performed in parallel. Hierarchical assembly trees can be designed in such a way that intermediates yield functional subunits which can be reused for later constructs.

We designed a minimal vector backbone (1.9 kb) based on pUC19 [8] comprising the *β*-lactamase gene conferring resistance to ampicillin and the pMB1 replicon harboring a single point mutation to achieve high copy replication of the plasmid. The minimal multiple cloning site (minimal MCS) of the empty backbone contains the bioinformatically identified restriction sites MluI, XhoI and SalI (an additional EcoRV site was added to the 5’-end of the MCS to enable cost-efficient analytical restriction digests). In this MXS- (**M**luI-**X**hoI-**S**alI-) chaining standard, each individual building block is flanked by MluI and XhoI sites on its 5’-end and a SalI site on its 3’-end (Fig 1A). Two such building blocks can be joined together in the desired order by isolating the block to form the 5’-part with an MluI-, SalI-double digest followed by ligation with the MluI-, XhoI-linearized vector containing the block to form the 3’-part. The SalI- and the XhoI-overhangs are compatible and will produce a translatable scar (translated into valine-glutamic acid) upon ligation. The original pattern of restriction sites is thus regenerated in the ligation product, which enables the reiteration of the chaining approach. It should be noted that MXS-chaining is directional, however the choice of restriction digests for individual blocks determines the direction of chaining.

Cutting any building block with MluI and SalI restriction enzymes will liberate the entire insert sequence from the minimal vector backbone. If the insert is of similar size as the minimal vector backbone (1.9 kb), we recommend adding a third restriction enzyme (either AseI, PvuI, ScaI or XmnI) that will cut inside the minimal vector backbone sequence. This facilitates isolating the insert by gel electrophoresis and its subsequent purification. It should however be ascertained that the recognition sequence of the chosen enzyme is not found within the respective insert sequence.

Table 1 lists a selection of building blocks and preassembled cassettes, which we provide to the community via the Addgene repository. In the following sections, we describe how to generate new customized building blocks for users who wish to expand this collection of building blocks to meet the requirements of their specific experimental questions.

**Table 1:** MXS-building blocks available from Addgene.

**Addgene ID**	**Name**	**Short Description**
**Fluorescent Proteins** with ATG start codon, no stop codon (excitation/emission maxima)		
62400	MXS_TagBFP	399 nm/456 nm [17]
62401	MXS_Cerulean	433 nm/475 nm [18]
62402	MXS_LSS-mKate2	460 nm/605 nm [19]
62403	MXS_mEGFP	488 nm/507 nm [20]
62404	MXS_mAzamiGreen	492 nm/505 nm [21]
62405	MXS_Citrine	516 nm/529 nm [22]
62406	MXS_mKO2	551 nm/565 nm [5]
62407	MXS_tdTomato	554 nm/581 nm [23]
62408	MXS_mCherry	587 nm/610 nm [23]
62409	MXS_mPlum	590 nm/649 nm [24]
62410	MXS_E2-Crimson	611 nm/646 nm [25]
62411	MXS_iRFP670	645 nm/670 nm [26]
62412	MXS_PA-GFP	504 nm/517 nm after photoactivation [27]
62413	MXS_Kaede	508 nm/518 nm and 572 nm/582 nm [28]
**Promoters and Enhancers**		
62414	MXS_PGK	PGK promoter [29]
62415	MXS_minPGK	minimal PGK promoter [30]
62416	MXS_PGKenhancer	PGK enhancer [30]
62417	MXS_CMV	CMV promoter [31]
62418	MXS_minCMV	minimal CMV promoter [31]
62419	MXS_CMVenhancer	508 nm/518 nm and 572 nm/582 nm [28]
62420	MXS_CAG	CAG promoter [32]
62421	MXS_EF1a	EF1*α* promoter [33]
62422	MXS_Tet	tetracycline-inducible promoter [6]
62423	MXS_BiTet	bidirectional tetracycline-inducible promoter [34]
**Polyadenylation Signals**		
62424	MXS_bGpA	*β*-Globin poly A [35]
62425	MXS_bGHpA	bovine growth hormone poly A [36]
62426	MXS_SV40pA	Simian virus 40 poly A [37]
**Tools for Inducible Gene Expression**		
62427	MXS_CreERT2	tamoxifen-inducible Cre recombinase [38]
62428	MXS_rtTA3	reverse tetracycline transactivator [39]
62429	MXS_tTA2	tetracycline transactivator [40]
**Miscellaneous**		
62430	MXS_loxP	loxP site
62431	MXS_linker	12 aa long Gly-, Ser-rich linker
62432	MXS_H2B	histone 2B with leading Kozak sequence
62433	MXS_P2A	self-cleaving peptide [41]
62434	MXS_PEST2D	destabilization tag [42]

Whenever possible, building block sequences should be generated by PCR from plasmids or cDNA. This allows the removal of unnecessary sequence parts and restriction sites and ensures the compatibility of coding sequences (Fig 1B). We recommend to design the template binding part of the forward and the reverse primers to reach a predicted melting temperature (OligoCalc [43]) of 60°C±1.5°C. The melting temperatures of pairs of primers should not differ by more than 1 K. We routinely add 5’-extensions to the template binding sequence of primers that harbor restriction sites for direct subcloning into the MXS-chaining context. The addition of these extensions did not adversely influence PCR efficiency or specificity in PCRs from cDNA, genomic or plasmid DNA. When cloning protein coding sequences, the downstream application of the building block should be considered. If the building block is either meant to contain the entire open reading frame (ORF) or is to be used strictly as the N-terminal part of an ORF, an optimized Kozak sequence [44] (5’-GCCGCCACC-3’) can be included into the 5’-extension of the forward primer immediately upstream of the start codon (ATG). If one wishes to preserve the possibility to use the respective building block as a C-terminal sequence part in protein fusions, the Kozak sequence should be omitted and the 5’-extension 5’-AATACGCGTAACTCGAG-3’ should be directly followed by the start codon (ATG) of the coding sequence. In most cases, it is advisable to exclude the stop codon from the building block design to be able to (re-)use it as the N-terminal part in protein fusions. The building blocks containing polyA signals (Table 1) include stop codons in all three ORFs. It should however be noted that omitting the stop codon will lead to the C-terminal addition of valine and glutamic acid. The 5’-extension 5’-CTAGTCGAC-3’ specifying a SalI restriction site is added to the sequence of the reverse primer.

If the respective insert sequence cannot be amplified by PCR, a chaining vector containing a suitable multiple cloning site (MCS) can be used for cut and paste cloning into the MXS-chaining context. We provide several MXS-chaining vectors with different multiple cloning sites (Table 2). If the compatibility of the respective insert’s ORF with the MXS-chaining standard is to be preserved, it might be necessary to design customized multiple cloning sites.

**Table 2:** Vectors compatible with the MXS-chaining strategy available from Addgene.

**Addgene ID**	**Name**	**Short Description**
62394	MXS_chaining vector	minimal EcoRV-**MluI**-**XhoI**-**SalI** MCS
62395	MXS_MCS1	EcoRV-**MluI**-**XhoI**-NheI-XbaI-NcoI-KpnI-BclI-BamHI-**SalI** MC
62396	MXS_MCS2	EcoRV-**MluI**-**XhoI**-EcoRI-BglII-KpnI-**SalI** MCS
62397	MXS_MCS3	**MluI**-**XhoI**-HindIII-PstI-**SalI** MCS
62398	MXS_MCS4	**MluI**-**XhoI**-PvuII-BglII-**SalI** MCS
62399	SMX_DEST vector	**SalI**-EcoRV-**MluI**-**XhoI** MCS for head-to-head arrangement of MXS blocks

All listed vectors carry an ampicillin resistance gene and a high-copy origin of replication. MCS: multiple cloning site.

Customized multiple cloning sites or other small building blocks (< 120 bp), such as localization or purification tags as well as recombination sites, can be generated using two complementary annealed oligonucleotides. These oligonucleotide pairs are designed to generate sticky ends mimicking an MluI-overhang at the 5’-end and a SalI-overhang at the 3’-end of the sequence, to enable direct cloning into the MXS-chaining context (Fig 1C). Specifically, an XhoI site and a short spacer (5’-TAACTCGAG-3’) have to be added to the 5’-end of the sense sequence, a single “G” has to be attached to its 3’-end to generate the double-stranded sequence part. The sequence of the forward primer is generated by adding the bases mimicking an MluI-overhang (5’-CGCG-3’) to the 5’-end of the double-stranded sequence part. The sequence of the reverse primer is yielded by attaching the bases mimicking a SalI-overhang (5’-TCGA-3’) to the 5’-end of the reverse complement of the double-stranded sequence part.

If MluI or SalI restriction sites are found within the insert sequence, the respective site should be removed by mutagenesis PCR prior to subcloning into the MXS-chaining context. If the sequence contains an XhoI site, we recommend to first subclone it via MluI-, SalI-digest into the MXS-chaining system and to mutagenize the internal XhoI site as a second step. This can be readily achieved for any coding sequence because of the redundancy of the genetic code. In cases where alterations of the original sequence might interfere with its function (e.g. enhancer function of noncoding genomic DNA), we recommend to incorporate into the construct layout a small customized multiple cloning site, which will be used to insert the respective sequence as a last step.

A particular advantage of chaining-based approaches is the possibility to quickly generate an array of repetitions of a specific module or sequence motif (Fig 1D). This asset proves valuable in fine-tuning the expression level of transcriptional reporters or artificial promoters by varying the number of repeats of a specific transcription factor binding motif.

Unlike standard restriction-based cloning procedures, chaining approaches allow to assemble individual subunits in parallel. It is advisable to design assembly trees yielding functionally relevant intermediates (e.g. selection cassettes) that are likely to be useful for the assembly of later constructs (Fig 1E). Table 3 lists a set of MXS-cassettes we provide to the community via the Addgene repository.

**Table 3:** MXS-cassettes available from Addgene.

**Addgene ID**	**Name**	**Short Description**
**Positive Selection Cassettes**		
62435	MXS_CMV∷HygroR-bGHpA	resistance against hygromycin B
62436	MXS_PGK∷HygroR-bGHpA	resistance against hygromycin B
62437	MXS_CMV∷NeoR-bGHpA	resistance against G418
62438	MXS_PGK∷NeoR-bGHpA	resistance against G418
62439	MXS_CMV∷PuroR-bGHpA	resistance against puromycin
62440	MXS_PGK∷PuroR-bGHpA	resistance against puromycin
62441	MXS_CMV∷ZeoR-bGHpA	resistance against zeocin
62442	MXS_PGK∷ZeoR-bGHpA	resistance against zeocin
**Tools for Inducible Gene Expression**		
62443	MXS_CMV∷CreERT2-bGHpA	tamoxifen-inducible Cre recombinase [38]
62444	MXS_PGK∷CreERT2-bGHpA	tamoxifen-inducible Cre recombinase [38]
62445	MXS_CMV∷rtTA3-bGHpA	reverse tetracycline transactivator [39]
62446	MXS_PGK∷rtTA3-bGHpA	reverse tetracycline transactivator [39]
62447	MXS_CMV∷tTA2-bGHpA	tetracycline transactivator [40]
62448	MXS_PGK∷tTA2-bGHpA	tetracycline transactivator [40]

### Efficiency and robustness of MXS-chaining

**Fig 2:**
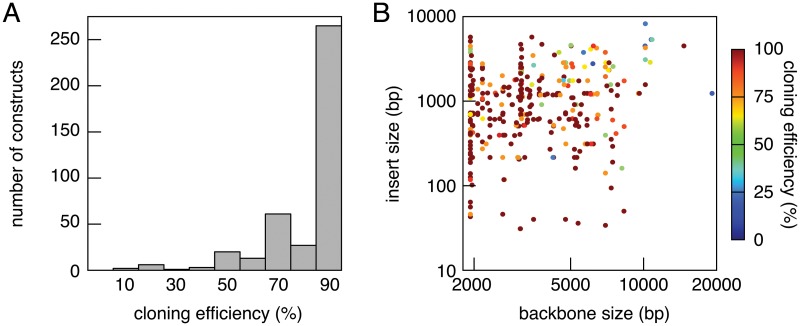
Efficiency of the MXS-chaining approach. (A) Cloning efficiency of the MXS-chaining approach measured as the fraction of positive recombinant plasmids resulting from MXS-chaining ligation reactions. (B) Dependence of the cloning efficiency on sizes of MX-digested vectors and MS-digested inserts. Data from 400 different MXS-chaining ligation reactions is shown.

Next, we assessed the efficiency of the MXS-chaining approach. MXS-chaining proved robust in assembling a wide range of constructs of up to 20 kb length and built from up to 34 individual building blocks. We analyzed by restriction digest the fraction of positive recombinant plasmids resulting from 400 ligation reactions. The cloning efficiency was excellent as two thirds of the reactions had an efficiency of over 90% (Fig 2A). Moreover 96% of the reactions yielded 50% or more positive transformants. In practice, based on the average cloning efficiency of 89.7%, the analysis of three colonies would give at least one positive transformant in 99.9% of the cases. Analyzing two colonies would yield at least one positive clone with a probability of 98.9%. We examined more closely if the cloning efficiency was dependent on the insert or vector size (Fig 2B). There was no particular correlation between insert or vector size and cloning efficiency.

### Applying MXS-chaining to generate constructs for single-cell experiments

Our objectives for the MXS-chaining system was the modular assembly of highly similar sequences (such as promoters, polyA signals or fluorescent proteins) into polycistronic constructs performing complex tasks in mammalian cell culture systems. To demonstrate the versatility of this approach, we will highlight examples of such MXS-chaining based constructs that allow the visualization of cellular architectures or processes in single cells by live-cell imaging or flow cytometry.

#### Constructs to label subcellular structures in live cells

A major application of fluorescent proteins (FPs) is the visualization of subcellular protein localization in living cells. To this end, synthetic constructs are transfected that express a protein of interest, which is fused in-frame to an FP. The variety of FPs with distinct spectral properties allows the simultaneous tagging of multiple proteins [45]. The use of polycistronic constructs for multi-color tagging ensures that the individual units are expressed at stoichiometric levels.

**Fig 3:**
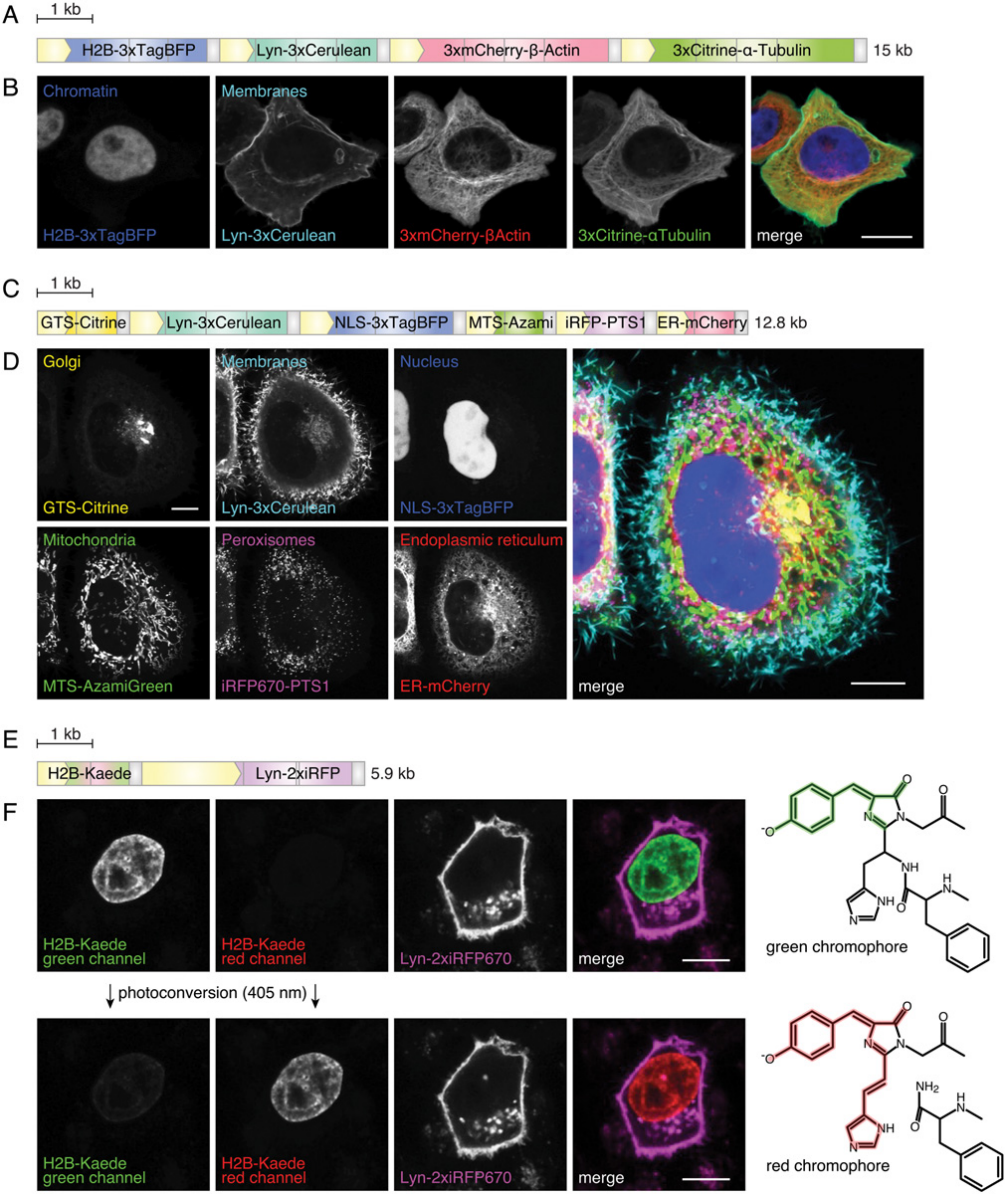
Tagging subcellular structures in live cells. (A) Scheme of the construct to label nuclei, the membranes and cytoskeleton components. CMV-promoters (yellow boxes) drive the expression of trimerized fluorescent proteins fused to histone 2B (H2B), Lyn-tag, *β*-Actin or *α*-Tubulin. Gray boxes are bovine growth hormone polyAs (bGHpAs). Scale bar: 1 kb. (B) Confocal section of HeLa cells expressing the construct shown in (A). Cell organelles (nucleus and membrane) and cytoskeleton components (Actin and Tubulin) are labeled. Scale bar: 10 *μ*m. (C) Scheme of the construct. CMV-promoters (yellow boxes) drive the expression of GTS-Citrine, Lyn-3xCerulean, NLS-3xTagBFP, MTS-AzamiGreen, iRFP670-PTS1 and ER-mCherry. Gray boxes are bGHpAs. GTS: Golgi-targeting signal, NLS: nuclear localization signal, MTS: mitochondrial targeting signal, PTS1: peroxisomal targeting signal 1, ER: N-terminal calreticulin signal peptide and C-terminal retention signal -KDEL. Scale bar: 1 kb. (D) Confocal section of HeLa cells expressing the construct shown in (C). Organelles labeled are: Golgi apparatus (Citrine), membrane (Cerulean), nucleus (TagBFP), mitochondria (AzamiGreen), peroxisomes (iRFP670) and endoplasmic reticulum (mCherry). Scale bar: 10 *μ*m. (E) Scheme of the construct. A CMV-promoter (small yellow box) drives the expression of H2B-Kaede and a CAG-promoter (large yellow box) drives the expression of Lyn-2xiRFP670. Small gray boxes are bGHpAs, large gray boxes *β*-Globin polyA (*β*-GpA) sequences. Scale bar: 1 kb. (F) Confocal section of HeLa cells expressing the construct shown in (E) before (top row) and after (bottom row) photoconversion by a 405 nm laser. The chromophore group structures in the green- and red-emitting states are depicted on the right (adapted from [49]). Scale bar: 10 *μ*m.

We designed a construct to label the cell nucleus, the membranes, the actin cytoskeleton and microtubules in human HeLa cells (Fig 3A and 3B). These structures were visualized using four FPs with minimal spectral overlap: TagBFP (excitation/emission maxima at 399 nm/456 nm), Cerulean (433 nm/475 nm), mCherry (587 nm/610 nm) and Citrine (516 nm/529 nm). Three copies of these FPs were fused in-frame (3xFP) to increase the brightness of the signal. An N-terminal fusion of histone 2B (H2B) to 3xTagBFP was used to tether this blue emitting FP to the chromatin. 3xCerulean was N-terminally fused to a Lyn-tag. This peptide targeting signal derived from the tyrosine-protein kinase Lyn contains a myristoylation and a palmitoylation site [46–48] and is used to anchor proteins to membranes. 3xmCherry was C-terminally fused to the full-length ORF of the human *β*-Actin (ENSG00000075624), 3xCitrine to the human *α*-Tubulin (ENSG00000123416). The human cytomegalovirus (CMV) promoter was used in each of the distinct units to drive high-level expression and the bovine growth hormone polyA signal (bGHpA) was used to terminate transcription. The individual expression units (MXS_*CMV∷H2B-3xTagBFP-bGHpA*, MXS_*CMV∷Lyn-3xCerulean-bGHpA*, MXS_*CMV∷3xmCherry-*β*-Actin-bGHpA*-S, MXS_*CMV∷3xCitrine-*α*-Tubulin-bGHpA*) were chained together to form a polycistronic construct with a 15 kb insert.

Subcellular structures are often visualized by fusing FPs to full-length ORFs of proteins known to localize to the structure of interest. It is hereby critical to notice that the observed phenotype in such an experiment might be influenced by the overexpression of the protein used to label the compartment. Deletion experiments have identified a number of short peptide targeting signals [50] that are sufficient to recapitulate the localization of the parental protein when fused to FPs. We made use of such known peptide targeting signals to label the Golgi apparatus (using a 25 aa Golgi targeting signal (GTS) from human Golgi phosphoprotein 2 (GOLPH2) [51]), the nucleus (using a 9 aa nuclear localization signal (NLS) [52]), the mitochondria (using the N-terminal 25 aa mitochondrial targeting signal (MTS) of COX4 from *S. cerevisiae* [53]), the peroxisomes (using the C-terminal amino acid sequence -SKL known as peroxisomal targeting signal 1 (PTS1) [54, 55]) and the endoplasmic reticulum (using the 17 aa ER-localization signal of human Calreticulin [56, 57] and the C-terminal retention signal -KDEL [58]) (Fig 3C and 3D). We reused the MXS_*CMV∷Lyn-3xCerulean-bGHpA* cassette relying on the 10 aa Lyn-Tag to label membranes. mAzamiGreen (492 nm/505 nm) and iRFP670 (645 nm/670 nm) were used in addition to the previously employed FPs to enable 6-color labeling. The short C-terminal peptide sequences -SKL and -KDEL were added as primer extensions in PCR reactions to the ORFs of the respective FPs. All other described peptide targeting signals were cloned using pairs of annealed oligos whose overhangs were compatible with the MXS-chaining system (Table 4).

**Table 4:** Annealed pairs of oligos for direct MXS-chaining.

**Name**	**Sequence**
CalrER_F	5’-CGCGTAACTCGAGATGCTGCTATCCGTGCCGTTGCTGCTCGGCCTCCTCGGCCTGGCCGTCGCCG-3’
CalrER_R	5’-TCGACGGCGACGGCCAGGCCGAGGAGGCCGAGCAGCAACGGCACGGATAGCAGCATCTCGAGTTA-3’
GTS_F	5’-CGCGTAACTCGAGATGAAGTCGCCGCCCCTCGTGCTGGCCGCCCTGGTGGCCTGCATCATCGTCTTGGGCTTCAACTACTGGATTGCGG-3’
GTS_R	5’-TCGACCGCAATCCAGTAGTTGAAGCCCAAGACGATGATGCAGGCCACCAGGGCGGCCAGCACGAGGGGCGGCGACTTCATCTCGAGTTA-3’
Lyn_F	5’-CGCGTAACTCGAGATGGGCTGTATCAAGAGCAAGAGGAAGGACGTCGAGAACG-3’
Lyn_R	5’-TCGACGTTCTCGACGTCCTTCCTCTTGCTCTTGATACAGCCCATCTCGAGTTA-3’
MTS_F	5’-CGCGTAACTCGAGATGCTGAGCCTGCGGCAGAGCATCCGGTTCTTCAAGCCCGCCACCCGGACCCTGTGCAGCAGCCGGTACCTGCTGG-3’
MTS_R	5’-TCGACCAGCAGGTACCGGCTGCTGCACAGGGTCCGGGTGGCGGGCTTGAAGAACCGGATGCTCTGCCGCAGGCTCAGCATCTCGAGTTA-3’
NLS_F	5’-CGCGTAACTCGAGATGGCTCCTAAGAAGAAGAGGAAGGTGG-3’
NLS_R	5’-TCGACCACCTTCCTCTTCTTCTTAGGAGCCATCTCGAGTTA-3’
tetO_F	5’-CGCGTAACTCGAGTTTACCACTCCCTATCAGTGATAGAGAAAAGTGAAAG-3’
tetO_R	5’-TCGACTTTCACTTTTCTCTATCACTGATAGGGAGTGGTAAACTCGAGTTA-3’

A major innovation in expanding the performance options of FPs was the discovery of Kaede as the first photoswitchable fluorescent protein [28]. Using standard microscopy equipment, Kaede can be readily switched from a green- to a red-fluorescence (508 nm/518 nm and 572 nm/582 nm) emitting state (Fig 3D and 3E). The irreversible photoconversion upon UV irradiation (405 nm) is accomplished by a *β*-elimination reaction extending the conjugated *π*-electron system of the chromophore [49] (Fig 3E, right panels). This feature proved valuable as it enabled scientists to selectively label individual cells. We therefore included Kaede into our toolbox of MXS-building block.

#### A FUCCI-based construct to monitor cell cycle progression in transgenic mouse embryonic stem cells

**Fig 4:**
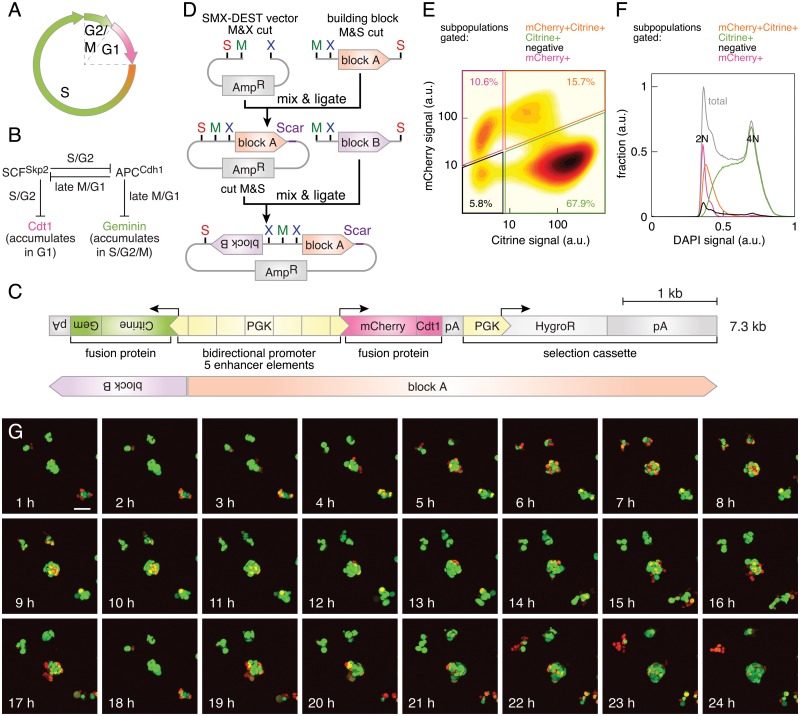
Adapting the FUCCI system to mouse embryonic stem cells. (A) FUCCI labels cells in G1 phase red, cells at the transition to S phase orange and cells in late S, G2 and M phases in green. (B) Molecular mechanism behind FUCCI. A double-negative feedback loop between the SCF^Skp2^ and APC^Cdh1^ complexes exhibits bistability and leads to the oscillating accumulation of their substrates Cdt1 in G1 phase and Geminin in late S, G2 and M phases (adapted from [5]). (C) Scheme of the FUCCI-based construct to monitor cell cycle progression in mESCs. Top: A bidirectional PGK-promoter containing five enhancer elements drives the expression of Citrine-Gem(aa 1–110) and mCherry-hCdt1(aa 30–120). Small gray boxes are bGHpAs, large gray boxes *β*-GpAs. Scale bar: 1 kb. Bottom: Orange and purple boxes indicate the identity of the building blocks, which were assembled head-to-head into the SMX_DEST vector, see as well (D). (D) Scheme of the cloning strategy using the SMX_DEST vector to arrange building blocks head-to-head. (E) Flow cytometry analysis of mCherry and Citrine fluorescence in a clonal transgenic mESC line stably expressing the construct shown in (C). The percentage of cells in each gate is shown. (F) Analysis of the cellular DNA content. DAPI signal distribution of each subpopulation outlined in (E) and of the total culture is shown. (G) Live imaging of a clonal transgenic culture expressing the construct shown in (C). Maximum intensity projections of confocal sections are displayed. Scale bar: 50 *μ*m.

The development of the fluorescent, ubiquitination-based cell cycle indicator (FUCCI) has enabled scientists to visualize the cell cycle transition of living cells from G1 to S phase [5] (Fig 4A). The system relies on the action of two E3 ubiquitin ligase complexes, which oscillate reciprocally during the cell cycle thereby tagging their substrates for degradation in a cell-cycle-dependent manner (Fig 4B). The SCF^Skp2^ complex is stabilized during S and G2 phases, whereas the APC^Cdh1^ complex is active during late M and G1 phases of the cell cycle. Fusing a green and a red fluorescent protein to partial sequences of Geminin (Gem), a ubiquitin-target of the APC^Cdh1^ complex, and hCdt1, a substrate of the SCF^Skp2^ complex, yielded an indicator marking the nuclei of cells in S/G2/M phases in green and G1 phase nuclei in red [5]. The original layout of the system was based on the cotransduction of two lentiviral vectors, in which the human EF1*α*-promoter drove the expression of mAzamiGreen-Gem(aa 1–110) and mKO2-hCdt1(aa 30–120).

We aimed to adapt this system to visualize cell cycle dynamics in mouse embryonic stem cells (mESCs). As retroviral vectors are subject to silencing in mESCs [59], we decided to create stably expressing cell lines by random integration of the transgene into the genome. We incorporated a positive selection cassette conferring resistance to hygromycin B (MXS_*PGK∷HygroR-*β*GpA*) into the construct layout to enable efficient selection of stable clones (Fig 4C). The choice of fluorescent proteins was adapted to our imaging platform by replacing mAzamiGreen (492 nm/505 nm) with Citrine (516 nm/529 nm) and mKO2 (551 nm/565 nm) with mCherry (587 nm/610 nm). Instead of the EF1*α*-promoter used in the original vectors, we based our design on the promoter of the murine *3-phosphoglycerate-kinase 1* (PGK-promoter)[29], which had been selected for its high expression in mESCs [60]. Our layout featured a bidirectional version of the PGK-promoter to achieve highly correlated transcription of Citrine-Gem(aa 1–110) and mCherry-hCdt(aa 30–120). Using a single PGK-enhancer [30] module between two minimal PGK-promoters did not yield any transgenic lines with sufficient transgene expression. However, five enhancer modules led to satisfying transgene expression in more than 85% of all clonal mESC lines analyzed. This design was implemented by subcloning the MluI-, SalI-digested cassette MXS_*5xPGKenhancer-PGKmin∷mCherry-hCdt1(30–120)-bGHpA-PGK∷HygroR-*β*GpA* into the MluI, XhoI-cut SMX_DEST vector (Table 2, Fig 4C and 4D). The product of this reaction was cut by MluI, SalI and ligated with the MluI-, SalI-digested MXS_*PGK min∷Citrine-Geminin(1–110)-bGHpA*.

We analyzed the DNA content of transgenic mESCs following DAPI staining by flow cytometry (Fig 4E and 4F). Strikingly, the mCherry^+^ subpopulation accounted for only 10.6% of the cells in the culture (Fig 4E), consistent with the shortened G1 phase observed in mESCs [61]. Importantly, examination of the DAPI signal distribution among mCherry^+^ indicated a 2N cellular DNA content confirming their G1 phase identity. mCherry^+^Citrine^+^ and Citrine^+^ subpopulations exhibited partial and partial to full genome replication. The non-fluorescent subpopulation split into 4N and 2N cells, and hence constituted dividing cells in M phase. Live imaging of clonal transgenic mESCs confirmed the oscillation between red and green signal in single cells over time (Fig 4G).

#### Constructs to titrate inducible transgene expression

**Fig 5:**
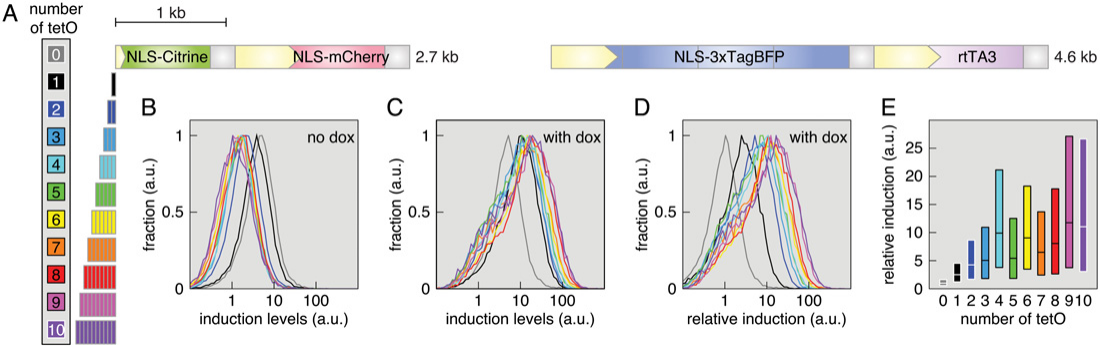
Varying the number of tetO sequences to titrate doxycycline-inducible transgene expression. (A) Scheme of the reporter constructs containing 0–10 tetO elements and the construct expressing the reverse tetracycline transcactivator (rtTA). Scale bar: 1 kb. (B) Distribution of reporter signal in uninduced (no doxycycline added) HeLa cells measured by flow cytometry. Lines are color-coded according to the legend depicted in (A). (C) Distribution of reporter signal in induced (doxycycline added 24 h before analysis) HeLa cells. (D) Distribution of reporter signal in induced HeLa cells normalized by the background levels for each reporter construct. (E) Box plot showing the normalized reporter signal for each reporter construct.

Transcriptional reporters containing multimerized response elements have long been used to dissect pathway responses [62, 63]. To clone long multimers, a single oligonucleotide containing the response element was generally allowed to self-ligate. Thus the number of repeats generated by this method could not be controlled and had to be determined *a posteriori*. Taking advantage of the flexibility offered by the MXS-chaining approach, we systematically varied the number of response elements using as a model the tetracycline-controlled gene expression system [6]. The tetracycline transactivator protein (tTA) binds tetO operator sequences in the absence of tetracycline. In their original paper, Gossen and Bujard observed a higher expression when 7 tetO sequences were used compared to one or two [6]. We used a reverse tetracycline transactivator protein (rtTA) that had been modified to bind tetO sequences upon addition of doxycycline, a tetracycline analog [39]. A fluorescent transcriptional reporter was constructed by chaining Citrine downstream of a minimal CMV-promoter (Fig 5A). The number of tetO sequences (Table 4) was systematically varied between one and ten and these multimers were chained in front of the minimal CMV-promoter. As transfection control, we incorporated into the same plasmid an mCherry expression cassette. HeLa cells were cotransfected with the tetO reporter plasmids and an rtTA-expressing plasmid (Fig 5A). We assessed reporter expression in single cells by flow cytometry 24 h after addition of doxycycline. Interestingly, background expression levels without doxycycline addition were decreased with increased number of tetO sequences (Fig 5B). Upon addition of doxcycline, we observed an increase in reporter signal dependent on the number of tetO sequences (Fig 5C). The response was normalized by the background levels for each reporter construct (Fig 5D). The median reporter expression increased with the number of tetO sequences and plateaued at an 11-fold induction for 9 and 10 tetO sequences (Fig 5E). Surprisingly, that increase was not monotonous as reporters containing 5 and 7 elements were less potent than reporters containing 4 and 6 tetO elements. This might be due to the fact that rtTA acts as a dimer [64] and even numbers of response elements might thus be preferred.

### Conclusion

To conclude, we have described a highly efficient cloning method for the assembly of constructs tailored to single-cell imaging or flow cytometry applications in mammalian cell culture systems. The bioinformatically identified enzyme combination (**M**luI, **X**hoI and **S**alI: MXS) enabled robust chaining of individual building blocks into large polycistronic constructs. A comprehensive set of MXS-building blocks comprising custom vectors, multiple fluorescent proteins, constitutive promoters, polyadenylation signals, selection cassettes and tools for inducible gene expression is available from the Addgene repository (https://www.addgene.org).
